# Scientific Evolution of Artificial Heart Valves: A Narrative Review

**DOI:** 10.7759/cureus.42131

**Published:** 2023-07-19

**Authors:** Tanishq Kumar, Arihant Singh, Swedaj Thakre, Sourya Acharya, Samarth Shukla, Sunil Kumar

**Affiliations:** 1 Medicine, Jawaharlal Nehru Medical College, Datta Meghe Institute of Higher Education and Research, Wardha, IND; 2 Pathology, Jawaharlal Nehru Medical College, Datta Meghe Institute of Higher Education and Research, Wardha, IND

**Keywords:** valves, heart, bioprosthetic, mechanical, medicine

## Abstract

Cardiovascular disorders have always been the top contributors to the number of mortality occurring worldwide. But the last few decades have seen a drop in those numbers as the lives of millions of people have been saved due to ground-breaking advances in both therapeutic and surgical treatment modalities. Achieving this level of scientific glory in cardiology was a challenging feat. The credit goes to the scientists and physicians of the previous century who, despite their time's technological limitations, made discoveries and laid a solid foundation for modern medicine. Valvular complications are a major part of the global burden of cardiac diseases. The ongoing development of heart valve replacements remains a fascinating subject, as it continues to progress. Valve replacements comprise either mechanical heart valves or bioprosthetic heart valves. Both types of valves have their merits and demerits; their usage depends mostly on individual patient requirements. This article aims to review the evolution of the implantation of heart valves, and it is the objective of this article to give credit to scientists and physicians for their contributions. The article highlights the research gaps in finding more durable materials and the scope of further research in creating a heart valve that can be universally used for better patient outcomes.

## Introduction and background

As per a report by the American Heart Association (AHA), pathological cardiac conditions form the bulk of diseases that contribute maximally to high numbers of deaths around the globe [1]. The genuine necessity to find novel treatment modalities involving both therapeutic and surgical interventions has dawned upon scientists and physicians for decades. The structure of the heart is quite complex and intriguing, and each component has a plethora of clinical significance associated with it.

The aortic valve is one of the four valves present in the heart. It is semilunar in shape and has three flap-like structures called cusps or leaflets. It is present between the aorta and left ventricle. Valves ensure the unidirectional flow of blood which plays a vital role in supporting systemic circulation. The three cusps are structurally developed to resist any abnormality in the fluid mechanics of the blood flow [2]. 

Several genetic or embryological defects regarding the development of heart valves contribute to various valvular complications. Some of the complications include aortic stenosis and aortic regurgitation. Aortic stenosis arises due to inadequate aortic valve opening, probably due to underlying processes like calcification. Aortic insufficiency or aortic regurgitation occurs when an abrupt loss of functionality of the aortic valve leads to the backflow of blood back towards the left ventricle [3]. Therefore, it is vital that the normal functionality of the heart valves should be maintained. Surgically, defects of the heart valves can be corrected either by implanting a mechanical or bioprosthetic heart valve. The choice, however, depends on the overall health of the patient and the patient's age.

Mechanical heart valves, on average, may normally function for up to 25 years. Although they are very durable, their biggest drawback is lifelong anticoagulation therapy. On the other hand, bioprosthetic heart valves have a short working span due to calcification but are hemodynamically more stable and are closest to natural valves [4-6].

**Figure 1:**
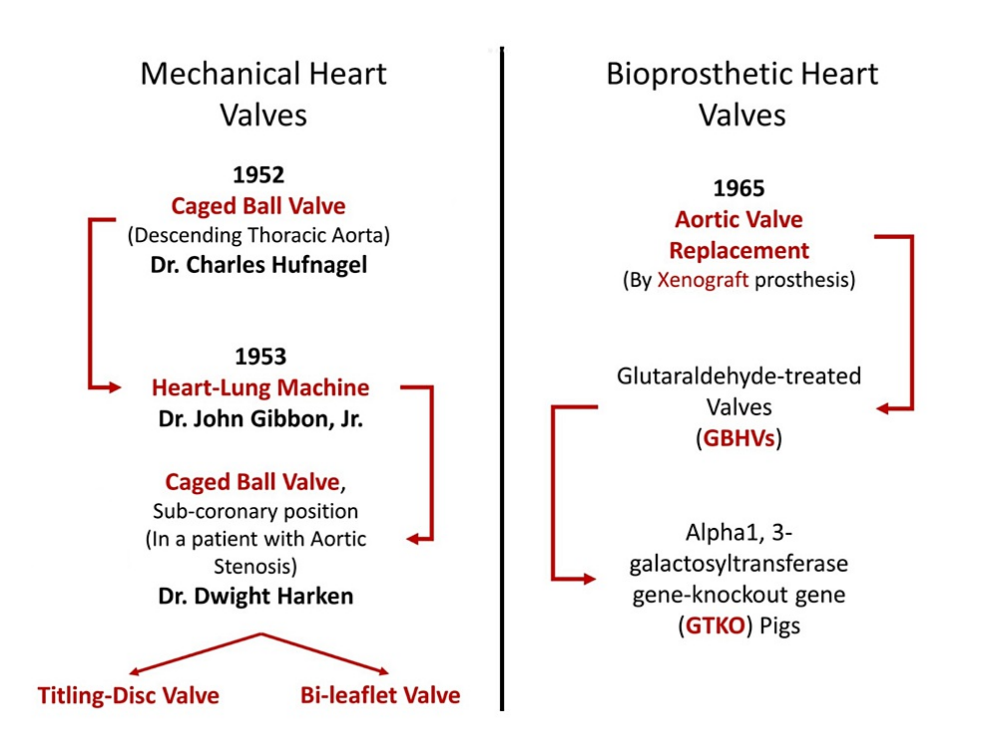
Evolution of Heart Valves (The figures are the authors' own creations.)

Surgically, the replacement of the damaged heart valve and implanting a new one was a basic answer to valvular pathology. However, the road to successful heart valve replacement was still a dream for the surgeons of that time. Figure 1 shows the evolution of heart valves.

## Review

Mechanical Heart Valves: Past

Several milestones and breakthroughs were achieved in the last century, especially in cardiac surgery. For example, a milestone in cardiac surgery was made possible in 1953 when Dr. John Gibbon Jr performed the world's first successful closure of an intracardiac defect and his invention of the heart-lung machine around the same time. Another milestone was achieved by Dr. Charles Hufnagel who was successful in implanting a caged ball valve in a patient with failed heart valves. The implantation did not prove beneficial to the patient because of its site of implantation and contributed minimally to the improvement of aortic insufficiency. Dr. Dwight Harken 1960 successfully implanted a caged ball valve in a patient with aortic stenosis. This time, the valve was implanted in a much more crucial site, the sub-coronary position. Since then, around 70 unique designs of prosthetic valves have been developed and implanted in many patients. It has been observed that mortality rates due to valve replacement have significantly improved since the 1960s. Out of all the designs and the engineering marvels of the mechanical heart valves, only six are still being used. They include Starr-Edwards aortic and mitral ball valves, the Omniscience tilting-disc valves, Omnicarbon tilting-disc valves, the Medtronic-Hall tilting disc valves, St. Jude and Carbomedic bileaflet valves. While the other designs of the valves may not be used today, their contribution to creating today's mechanical valves cannot be ignored [7-10].

Mechanical Heart Valves: Present

In today's time and age, two types of mechanical valves, i.e. tilting-disc and bileaflet valves, are being used. They have shown to have significant durability compared to their previous models. They, however, require a long-term anticoagulation regimen as they induce thrombotic and inflammatory responses to the implanted valves [11,12].

The greatest advantage of using mechanical valves is their durability. They are far more durable than bioprosthetic valves. Mechanical valves function remains stable for normally around ten more years than bioprosthetic valves [13-16]. Since they are more durable, reoperation rates are less than bioprosthetic heart valves [17].

Although mechanical valves are more durable, blood flowing around the valves causes high sheer stress, which in turn, induces the activation of platelets. This might lead to thrombosis on the valvular structure, further worsening the valves' condition and eventually increasing the chance of embolism. Therefore, mechanical heart valve implantation requires long-term anticoagulant therapy. Warfarin, a vitamin K antagonist, is most widely used in anticoagulation regimens. Now, there are two sides to warfarin use, it reduces the chances of thrombosis but increases the hemorrhagic risk [16-19]. Taking an example of a 60-year-old male patient, if a mechanical heart valve is used, it poses a 41% chance of bleeding compared to just a 12% chance of bleeding if a bioprosthetic valve had been used [20]. Also, it is hard to maintain the required dose of warfarin due to both barriers to adherence and the chemical reactions between warfarin and other dietary substances [21].

Another factor that hinders the adoption of mechanical heart valves is chronic illness from which the patient might suffer. For example, if a patient is already suffering from atrial fibrillation or thrombotic disease and is under anticoagulant therapy, heart valve implantation would be difficult. Research and studies are still needed for innovation in mechanical heart valves to address the issues of anticoagulation prescriptions, co-morbid conditions in patients, and more durable structure designs [22,23]. 

Mechanical Heart Valves: Future

In the last few decades, exceptional advances have been made in mechanical heart valves. Most of the development occurred based on the data derived from patient outcomes, and scientists are still trying to engineer more durable heart valves. The aim for the future is to create well-engineered, appropriate valves suited to each patient's need. Future designs should be of such material that can sustain without an anticoagulation regimen [7]. These changes will surely increase the life expectancy of patients with mechanical heart valves. Healthcare professionals should also formulate protocols that assess the possibilities of any side effects if a mechanical heart valve is being selected for implantation. Co-morbid conditions, pregnancies, and other chronic illnesses should also be considered when choosing between mechanical and bioprosthetic heart valves [11]. 

Bioprosthetic Heart Valves: Past

It was in Paris in the year of 1965 when Carpentier and colleagues achieved the feat of performing the world's first successful aortic valve replacement by a xenograft prosthesis [24]. Within the next three years, they conducted 61 more replacements in 53 patients but faced a low success rate. Around 45% of the newly implanted porcine valves showed disturbances in their functioning within one year. The failure rate could be due to structural or surgical reasons, as the scientists were limited by the technologies of that time. But Carpentier's achievement of xenograft replacement of human heart valves intrigued researchers worldwide [25]. According to a study by Talbert and Wright, there was evidence of rejection of bioprosthetic heart valves by the immune system [26]. This was followed by various studies attempting to find the reason for this immune response for the new implants and find ways to improve their success rate.

**Figure 2:**
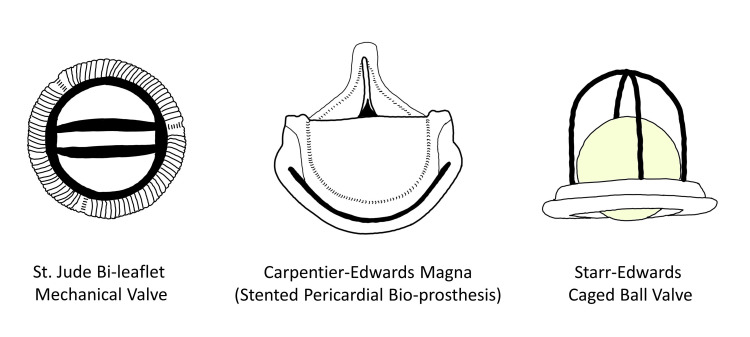
An Illustration of Older Heart Valves (The figures are the author's own creations.)

It was found that if the structural glycoproteins are denatured it would significantly lower the antigenicity of the porcine heart valves and help preserve the valves. This was done by treating the valves with glutaraldehyde which lowered the immune response. Glutaraldehyde-treated valves faced lower immunogenicity; therefore, close to 80% of valves did not lose efficacy in the first year compared to 45% earlier [25]. This milestone led to the large-scale use of glutaraldehyde-treated bioprosthetic heart valves (GBHVs) in patients who needed heart valve replacements. As more and more GBHVs were implanted, it was assessed, based on collected medical data, that GBHVs were becoming less potent with time. This time, the reason was calcification [27]. Figure 2 is an illustration of older heart valves.

Bioprosthetic Heart Valves: Present

The advent of GBHVs was instrumental in improving the care of patients with valve defects, but the issue of calcification was a huge obstacle. Chemical interactions between valves and blood (functional groups like free aldehyde group and phospholipids on valves react with calcium ions in the blood) lead to calcification [28].

Another factor that might contribute to calcification is structural valve deterioration (SVD). SVD is a function of time. There is calcification of the valve, and structural deterioration leads to a smaller aperture of the valve, disrupting the flow of blood. There might also be damage/breakage in the valve, defeating the entire purpose of the implantation of GBHV. Some studies recognize the link between inflammation and calcification, which might also contribute to calcification in GBHVs. Diseases causing chronic inflammation, like tuberculosis and atherosclerosis, might also contribute to the calcification of the GBHVs [29,30]. Based on scientific data, it has been found that less than 10% of GBHVs fail during the first ten years in patients with age more than 65 years. Whereas in comparatively young populations less than 35 years of age, GBHVs fail in the first five years [31]. This conclusion can be derived from the fact that the immune responses of two age groups have different potency of immunity which might contribute to two aspects of the process of calcification: The metabolism of free-flowing calcium ions in the blood and the immune response to the xenograft. Thus, it can be said that these bioprosthetic heart valves (BHVs) failed due to inflammation, and thrombosis and also showed evidence of histopathological findings similar to experimental xenotransplants [28,32,33].

On a molecular level, the failure arises due to the presence of galactose alpha 1,3 galactose (Gal) antigen found in pig tissues [34]. This antigen is the main target of various immune cells and leads to the antigen-antibody reaction, further culminating in calcification and loss of efficiency of BHVs. The solution to this problem could be the employment of genetically engineered Gal-deficient pigs providing bioprosthetic heart valves that do not succumb to immune responses and calcification [35,36]. 

Bioprosthetic Heart Valves: Future

Normally, there are not many complications in patients with BHV implants (derived from porcine), and they survive without anticoagulation. It is only in the younger populations who have significant structural deterioration with time demanding another implantation surgery [37]. Therefore, it is the need of the hour to focus more on BHVs suitable for the younger population. In the future, genetically modified pigs with no Gal antigen should be used for implanting valves in order to avoid calcification. These genetically modified pigs, known as alpha 1, 3-galactosyltransferase gene-knockout (GTKO), will possess genes that won't induce inflammation and thrombosis on the implantation of valves derived from them. Adopting GTKO pigs for BHVs would undoubtedly increase the cost of the entire heart valve implantation, which is already considered an expensive surgery in developing countries.

**Figure 3:**
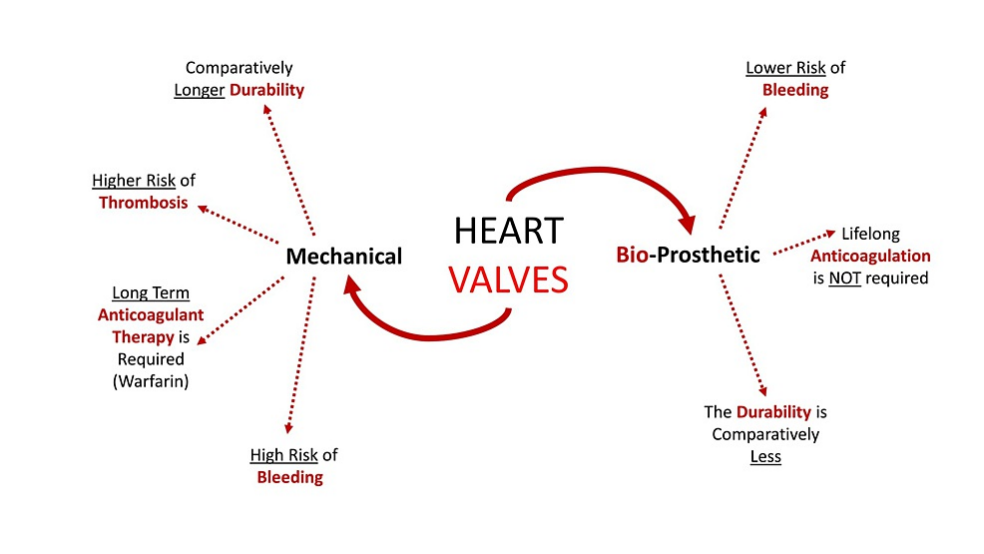
Differences between Mechanical and Bioprosthetic Heart Valves (The figures are the author's own creations.)

Thus, for another breakthrough in the field of bioprosthetic heart valves, innovation is needed to make the surgery cheap and within reach of the masses, durable for younger patients and patients that cannot be put in a long-term anticoagulation regimen [37]. Figure 3 shows the differences between mechanical and bioprosthetic heart valves.

Indian Heart Valves

In the Indian scenario, the cost of heart valves plays a huge limiting factor for the patients to access cardiac care. Keeping in mind the cost barrier, Chitra tilting disc valve was developed. The cost of a Chitra heart valve is comparatively lower than other valves available in the Indian market [38]. Since its development, more than 100,000 Chitra valves have been implanted. Based on a study, it has been concluded that the Chitra heart valve has low side effects and remains safe and functional even after 15 years of implantation [39].

Pricing of Heart Valves

**Figure 4:**
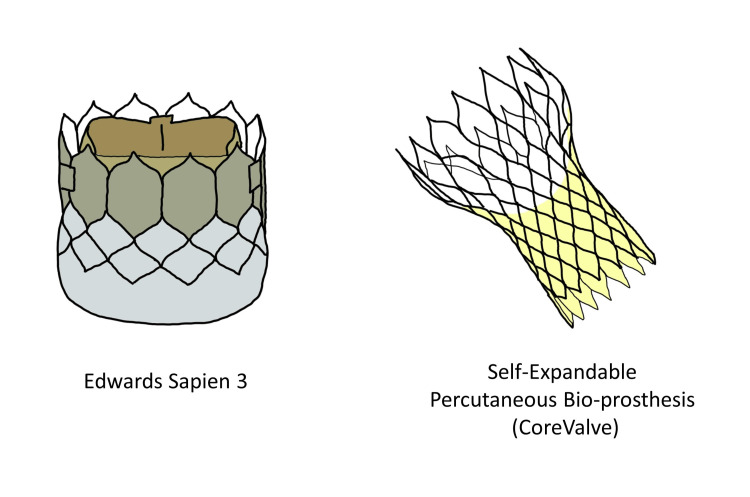
An Illustration of Newer Heart Valves (The figures are the author's own creations.)

According to a report by one of the leading institutes in India, the average cost of treating valvular heart diseases by valve implantation is estimated to be around INR100,000 to INR160,000. Mechanical and bioprosthetic valves cost around INR107,800 (US$1684) and INR154,000 (US$2406), respectively. More research is required for creating valves that are financially feasible for the masses [40]. Figure 4 illustrates newer heart valves.

Recent Developments

Transcatheter aortic valve implantation (TAVI), first performed in 2002, has become a standard option for treating aortic stenosis and mainly for younger patients [41]. Transcatheter mitral valve replacement (TMVR) seems promising in the treatment of mitral regurgitation but is still associated with complications like device dislocation and thrombosis [42].

With constant research in providing better cardiac care, there is an increased focus on treating tricuspid regurgitation. Transcatheter techniques relying on annulus structural changes, repairing impaired leaflet coaptation, lowering blood flow return to the vena cava, and implantation of valves are being developed. Some devices used for treating tricuspid regurgitation are Cardioband, Mitraclip, and Tric valves [43].

**Figure 5:**
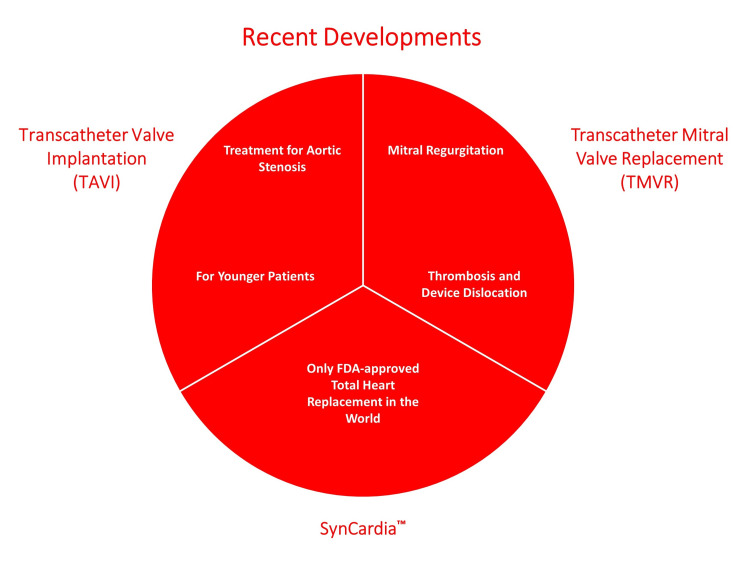
An Illustration of Recent Developments in Heart Valves (The figures are the author's own creations.)

The development of a total artificial heart (TAH) known as The SynCardia™ is a scientific wonder and a feat that humankind could never think of achieving just 50 years ago. Pneumatically driven and competent enough to blood flows of more than 9 liters/min is a life-saving invention that has already been implanted in more than 1,000 patients needing a biventricular replacement device. It is the only FDA-approved TAH in the world. TAH is mostly being used in conditions of terminal biventricular heart failure along with ischemic or non-ischemic dilated cardiomyopathy [44]. Figure 5 highlights recent developments in heart valves.

## Conclusions

Significant advancements in replacing heart valves in the last few decades have contributed to achieving low mortality rates worldwide. Most of this success has resulted from trial and correcting errors to get better designs and formulations. The contributions made by Dr. Hufnagel and Dr. Harken have been fundamental in creating the mechanical heart valves that we use today. There is always the need to create more compatible heart valves that will not require lifelong anticoagulants.

Similarly, bioprosthetic heart valves have seen remarkable progress in the last few decades. Constant research in creating bioprosthetic heart valves from genetically engineered pigs that will undergo less valve deterioration is the need of the hour. Achieving this would allow the implantation of BHVs in younger and older patients. Scientists should focus on creating new designs and finding more durable materials in the future, which could improve the current status of heart valve replacements.
